# An investigation of lifestyle behaviors, mental well-being, and work-related outcomes among female teachers: mixed-methods evidence for occupational health promotion

**DOI:** 10.3389/fpubh.2026.1993916

**Published:** 2026-10-22

**Authors:** Shereen S. AlSuwaimel, Ali Saad R. Alsubaie

**Affiliations:** Department of Public Health, College of Public Health, Imam Abdulrahman Bin Faisal University, Dammam, Saudi Arabia

**Keywords:** absenteeism, female school teachers, job satisfaction, life satisfaction, lifestyle behaviors, mental well-being, occupational health, Saudi Arabia

## Abstract

**Background:**

Teachers’ health and quality of life are important to school health and education policy. This study examined lifestyle behaviors, mental well-being, satisfaction, and health-related absence among female schoolteachers in Saudi Arabia.

**Methods:**

A mixed-methods study in the Eastern Province combined a cross-sectional questionnaire completed by 562 female government-school teachers with semi-structured interviews with 19 teachers. Mental well-being was assessed using the WHO-5. Associations were examined using Spearman correlation and multivariable logistic regression. Interviews were analyzed using reflexive thematic analysis with an inductive approach.

**Results:**

Poor mental well-being (WHO-5 ≤ 50) was reported by 58.4% of teachers, and 24.4% scored ≤28. No moderate-intensity activity was reported by 42.3%; 77.4% of 561 respondents slept six hours or less. Unhealthy foods were consumed several times per week or almost daily by 52.5%; only 29.9% consumed fruit and vegetables daily. Poor or very poor sleep quality was reported by 19.4%, and tobacco use by 3.4%. Overall, 27.9% brushed their teeth less than twice daily, and 52.1% last visited a dentist over two years earlier. Tooth or gum pain at least sometimes during the preceding three months was reported by 61.9%. Sleep quality and duration showed moderate positive correlations with well-being (*r* = 0.390 and 0.302, respectively; *p* < 0.001). Physical activity, dietary behaviors, toothbrushing, and oral pain were also associated with well-being. Higher teaching load was weakly associated with lower satisfaction (*r* = −0.098, *p* = 0.020). Lifestyle and satisfaction scores remained associated with good well-being after adjustment (OR = 1.051, 95% CI: 1.030–1.073; and 1.035, 95% CI: 1.022–1.048, respectively). Self-rated health also remained associated with good well-being. Lower lifestyle and well-being scores were associated with more health-related absence (all *p* < 0.05). Five themes described demanding workdays, barriers to healthy routines, emotional strain, work extending into home life, and occupational health concerns.

**Conclusion:**

Unhealthy daily routines and poor mental well-being were common among teachers. These findings support greater attention to teacher health in education policy. School health promotion should include manageable work demands, time for rest, and access to preventive care. Sustained investment in workplace health promotion may support teachers’ well-being and quality of life.

## 1 Introduction

Teachers’ health is an occupational and public health concern. Teaching involves sustained interpersonal responsibilities, heavy workloads, and demands that can affect well-being (1). Lifestyle behaviors and working conditions are associated with teachers’ psychosocial health (2). Workplace health promotion (WHP) should include supportive working conditions and opportunities for healthy behaviors (3). This approach is relevant to Saudi Arabia’s Vision 2030 priorities for healthy lifestyles and population well-being (4).

Saudi studies have reported depressive symptoms among primary-school teachers in Dammam, Khobar, and Qatif and psychological distress associated with work burden and limited support in Jeddah (5, 6). Research elsewhere also documents teachers’ psychological distress and burnout (7, 8). Burnout has been examined in relation to work ability, absenteeism, job satisfaction, and parental disengagement (9–11).

Physical activity, diet, tobacco use, and sleep are relevant to educators’ occupational health (12, 13). Physical activity and sleep have been examined in relation to burnout and psychological health (14–16), while insomnia has been documented among Saudi teachers (17). Earlier regional studies reported lower smoking prevalence among female teachers than male teachers (18, 19). Saudi studies have also examined teachers’ oral health knowledge, literacy, and attitudes (20, 21).

Lifestyle behaviors should also be considered together. Research among Portuguese teachers and Japanese employees links lifestyle measures with perceived stress or depressive symptoms (22, 23). Work demands and working hours may constrain healthy routines (24, 25). Saudi research has examined teachers’ quality of life and job satisfaction, workplace relationships, and faculty resilience (26–28). Musculoskeletal problems add to teachers’ health burden (29–31). These findings support considering health behaviors alongside working conditions and occupational outcomes.

Teacher health is also part of comprehensive school health. The Centers for Disease Control and Prevention (CDC) includes employee wellness among the ten components of its Whole School, Whole Community, Whole Child (WSCC) framework. This component recognizes schools as workplaces and supports staff physical and mental health, with potential benefits for student health and learning (32). Teachers can model healthy behaviors and help create supportive learning environments. Policies that expect teachers to promote student health should also provide conditions that protect their own health. Better teacher well-being has been associated with better student well-being and lower student psychological distress (33).

The World Health Organization (WHO) and United Nations Educational, Scientific and Cultural Organization (UNESCO) jointly developed health-promoting school standards that address the health of students, staff, and communities (34). From a population health perspective, schools offer a setting for prevention across generations. Sustained investment in teacher health should therefore be considered within education and health policy, with teachers’ quality of life as a goal in its own right. Organizational support includes school leaders’ commitment to staff health and opportunities to participate in wellness activities. A multilevel study linked perceived management support and supportive leadership with healthier teacher lifestyles (35).

Evidence examining lifestyle behaviors and mental well-being together among female teachers in the Eastern Province remains limited. This mixed-methods study assessed lifestyle behaviors and mental well-being among female school teachers in this region. It examined their associations with satisfaction and absenteeism. Interviews explored teachers’ experiences of work and health.

## 2 Materials and methods

### 2.1 Study design and setting

The study used a mixed-methods design, combining a cross-sectional, self-administered questionnaire with semi-structured interviews. Participants were female teachers employed in government schools in the Eastern Province of Saudi Arabia.

### 2.2 Participants and sampling

The target population comprised 21,442 female government-school teachers registered with the Eastern Region Education Directorate. The required sample size was 378, calculated using Cochran’s formula with finite population correction, a 95% confidence level, and a 5% margin of error. The planned sample was proportionately allocated across primary (n = 190), intermediate (n = 93), and high school (n = 95) levels using stratified random sampling. Of 756 teachers approached, 562 participated (74.3%). For the interviews, 19 teachers were randomly recruited. Eligible participants were practicing female teachers in government schools in the Eastern Province who consented to participate. The 19 interviews provided context for survey findings within a defined occupational group. They were not intended to estimate theme prevalence. Saturation is not used here to justify the interview sample, consistent with reflexive thematic analysis (36).

### 2.3 Instruments and variables

The questionnaire collected sociodemographic and clinical information, including age, marital status, educational level, teaching level, school location, weekly workload, chronic disease status, body weight category, teaching experience, and self-rated overall health. An 11-item lifestyle index covered five domains: physical activity, diet, sleep, tobacco use, and oral health. Ordinal item scores were summed (range 0–36). The 0–100 score was calculated as 100 × the raw sum / 36, with higher scores indicating healthier behaviors. Mental well-being was assessed using the Arabic WHO-5 Well-Being Index, which measures well-being over the preceding two weeks (37). The five item scores (0–5 each) were summed and multiplied by four to give a 0–100 score (37). Scores ≤50 indicated poor well-being, and scores ≤28 indicated very low well-being consistent with a positive depression screen (38). These thresholds do not establish a clinical diagnosis. Satisfaction was measured using eight items with six response categories (Supplementary Table S3). The items covered work capacity, work-life balance, work environment, professional relationships, administrative support and recognition, professional development, overall life satisfaction, and current job satisfaction. The reported composite ranged from 0 to 100, with higher scores indicating greater satisfaction. A semi-structured interview guide explored teachers’ experiences of workload, lifestyle, and well-being. Fatigue refers to interview participants’ descriptions of tiredness or exhaustion. It was not measured as a separate quantitative outcome using a validated fatigue scale.

### 2.4 Reliability of the study instruments

Cronbach’s alpha was 0.920 for the WHO-5 Well-Being Index and 0.853 for the satisfaction scale. Internal consistency was also assessed for the lifestyle index.

### 2.5 Procedure and data collection

Population data were obtained from the Eastern Region Education Directorate. Teachers were recruited by teaching level using proportional stratified random sampling. The 19 teachers were interviewed individually. Interviews were transcribed and organized for coding.

### 2.6 Statistical and qualitative analysis

Analyses were performed using IBM SPSS Statistics, version 29. Distributions were assessed using the Shapiro–Wilk test and Q-Q plots. Categorical variables were summarized as frequencies and percentages, and composite scores as medians and interquartile ranges (IQRs). Kruskal-Wallis tests compared scores across participant groups. Spearman correlations assessed associations between ordinal variables and composite scores. Variables meeting the screening threshold of *p* < 0.2 in univariable logistic regression were entered into a multivariable model of good mental well-being (WHO-5 > 50). Cronbach’s alpha assessed internal consistency. Tests were two-tailed. Reported *p* values are unadjusted for multiple comparisons; *p* < 0.05 was the nominal threshold. The association analyses should therefore be interpreted as exploratory. Interviews were analyzed using Braun and Clarke’s (39) reflexive thematic analysis with an inductive approach. Codes and themes were developed from teachers’ accounts, resulting in five themes. The adjusted model in Table 1 contains four variables represented by eight coefficients, excluding the intercept. Chronic disease count and self-rated health contribute two and four coefficients, respectively. Lifestyle and satisfaction scores each contribute one.

**Table 1:** Univariable and multivariable logistic regression of factors associated with good mental well-being (WHO-5 > 50).

Variable	Univariable OR (95% CI)	Univariable *p*	Multivariable OR (95% CI)	Multivariable *p*
Age (years), overall		0.738		
20–29	reference			
30–39	0.765 (0.268–2.183)	0.616		
40–49	0.591 (0.220–1.585)	0.296		
50–59	0.608 (0.225–1.642)	0.326		
≥ 60	0.680 (0.206–2.246)	0.527		
Marital status, overall		0.317		
Single	reference			
Married	1.707 (0.904–3.221)	0.099		
Widowed	1.322 (0.540–3.240)	0.541		
Divorced	2.061 (0.721–5.888)	0.177		
Educational level, overall		0.791		
High school or less	reference			
Diploma	1.467 (0.261–8.228)	0.663		
Bachelor	0.936 (0.207–4.228)	0.932		
Postgraduate studies	0.889 (0.163–4.853)	0.892		
Teaching level, overall		0.721		
Primary school	reference			
Intermediate school	0.894 (0.597–1.339)	0.587		
High school	0.852 (0.559–1.299)	0.458		
Place of residence, overall		0.370		
School location, overall		0.347		
Teaching load (classes/week), overall		0.890		
< 20 classes	reference			
20–24 classes	1.028 (0.715–1.479)	0.880		
> 24 classes	1.279 (0.471–3.469)	0.629		
Chronic diseases, overall		0.003		0.679
None	reference		reference	
One	0.591 (0.401–0.871)	0.008	0.913 (0.581–1.436)	0.695
≥ 2	0.468 (0.260–0.845)	0.012	0.742 (0.374–1.472)	0.393
Body weight, overall		0.273		
Severely underweight	reference			
Underweight	0.750 (0.048–11.648)	0.837		
Normal weight	1.744 (0.156–19.487)	0.651		
Overweight	1.241 (0.111–13.855)	0.861		
Severely obese	1.091 (0.081–14.664)	0.948		
Teaching experience, overall		0.369		
< 5 years	reference			
5–9 years	1.046 (0.463–2.360)	0.914		
10–14 years	0.613 (0.335–1.124)	0.113		
15–20 years	1.017 (0.527–1.965)	0.959		
> 20 years	0.835 (0.499–1.399)	0.494		
Overall health rating, overall		<0.001		<0.001
Poor	reference		reference	
Fair	2.681 (0.586–12.265)	0.204	1.603 (0.320–8.046)	0.566
Good	4.295 (0.967–19.086)	0.055	1.927 (0.394–9.423)	0.418
Very good	11.687 (2.620–52.125)	0.001	3.703 (0.748–18.340)	0.109
Excellent	28.588 (6.012–135.944)	<0.001	7.038 (1.302–38.031)	0.023
Lifestyle index (per 1-unit increase)	1.080 (1.060–1.100)	<0.001	1.051 (1.030–1.073)	<0.001
Satisfaction score (per 1-unit increase)	1.052 (1.040–1.064)	<0.001	1.035 (1.022–1.048)	<0.001

The researchers’ public health background and interest in teacher health informed their attention to working conditions and daily routines. This perspective may have shaped how accounts of workload and health were interpreted. Themes represent an interpretation of participants’ accounts rather than an objective classification (40). Survey findings and interview accounts were compared during interpretation and reporting (41). Section 3.3 brings these findings together around sleep and daily routines, satisfaction, and health-related absence. This comparison provides context rather than independent confirmation. Member checking is not claimed as a validation procedure; it is not a universal requirement of reflexive thematic analysis (40).

Item-level response totals varied. Supplementary Table S1 contains 561 responses for sleep duration and 519 for physical activity duration, compared with 562 participants overall. Percentages for these items use the corresponding response totals. Tobacco quantity is reported for the 19 tobacco users. Each WHO-5 item and each of the eight satisfaction items has 562 responses in Supplementary Tables S2, S3.

### 2.7 Ethical considerations

Ethical approval was obtained from the Institutional Review Board of Imam Abdulrahman Bin Faisal University (IRB No. IRB-PGS-2025-03-0781; 9 November 2025). Administrative approval was obtained from the General Directorate of Education in the Eastern Province (Approval Letter No. 4700486596; 30 December 2025). Voluntary informed consent was obtained from all survey and interview participants.

## 3 Results

The study included 562 female teachers, of whom 435 (77.4%) were aged 40–59 years, 454 (80.8%) were married, and 509 (90.6%) held a bachelor’s degree. Half taught in primary schools (50.2%), and 65.3% taught 20–24 classes per week. At least one chronic disease was reported by 223 (39.7%) teachers, and 142 (25.3%) rated their health as poor or fair. Half were overweight (50.2%), and 44.3% had normal body weight (Table 2).

**Table 2:** Sociodemographic and clinical characteristics of school teachers and their association with lifestyle index scores.

Characteristic	*N* (%)	Lifestyle index, Median (IQR)	Kruskal-Wallis *p*	Spearman correlation	Spearman *p*
Overall	562 (100.0)	52.8 (45.1 to 61.1)			
Age (years)					
20 to 29	17 (3.0)	55.6 (41.7 to 63.9)	0.060	0.111	0.008
30 to 39	80 (14.2)	52.8 (44.4 to 58.3)			
40 to 49	238 (42.3)	51.4 (44.4 to 61.1)			
50 to 59	197 (35.1)	55.6 (47.2 to 63.9)			
≥ 60	30 (5.3)	58.3 (50.7 to 63.9)			
Marital status					
Single	49 (8.7)	50.0 (41.7 to 61.1)	0.348		
Married	454 (80.8)	52.8 (47.2 to 61.1)			
Widowed	38 (6.8)	50.0 (44.4 to 60.4)			
Divorced	21 (3.7)	52.8 (41.7 to 63.9)			
Highest educational level					
High school or less	7 (1.2)	52.8 (50.0 to 55.6)	0.583	−0.002	0.953
Diploma	21 (3.7)	50.0 (38.9 to 58.3)			
Bachelor	509 (90.6)	52.8 (47.2 to 63.9)			
Postgraduate studies	25 (4.4)	50.0 (44.4 to 61.1)			
Current teaching level					
Primary school	282 (50.2)	52.8 (44.4 to 61.1)	0.260	−0.013	0.754
Intermediate school	148 (26.3)	55.6 (47.2 to 63.9)			
High school	132 (23.5)	52.8 (44.4 to 61.1)			
Place of residence					
AlAhsa	5 (0.9)	47.2 (41.7 to 50.0)	0.007		
AlKhafji	12 (2.1)	47.2 (41.0 to 57.6)			
Dammam	153 (27.2)	50.0 (44.4 to 58.3)			
Hafr Al Baten	8 (1.4)	52.8 (49.3 to 65.3)			
Jubail	46 (8.2)	54.2 (47.2 to 63.9)			
Khobar	58 (10.3)	50.0 (44.4 to 58.3)			
Nairiya	12 (2.1)	51.4 (47.2 to 61.8)			
Qarya AlUlia	1 (0.2)	36.1 (36.1 to 36.1)			
Qatif	266 (47.3)	55.6 (47.2 to 63.9)			
Ras Tanura	1 (0.2)	52.8 (52.8 to 52.8)			
School location					
AlKhafji	11 (2.0)	47.2 (41.7 to 59.7)	0.247		
Dammam	132 (23.5)	50.0 (44.4 to 61.1)			
Hafr Al Baten	8 (1.4)	52.8 (49.3 to 65.3)			
Jubail	43 (7.7)	52.8 (47.2 to 63.9)			
Khobar	66 (11.7)	52.8 (44.4 to 58.3)			
Nairiya	13 (2.3)	50.0 (44.4 to 61.1)			
Qarya AlUlia	1 (0.2)	36.1 (36.1 to 36.1)			
Qatif	284 (50.5)	55.6 (47.2 to 63.9)			
Ras Tanura	4 (0.7)	54.2 (52.8 to 58.3)			
Teaching load (classes per week)					
< 20 classes	178 (31.7)	55.6 (47.2 to 63.2)	0.480	−0.048	0.260
20 to 24 classes	367 (65.3)	52.8 (44.4 to 61.1)			
> 24 classes	17 (3.0)	50.0 (41.7 to 61.1)			
Chronic diseases					
None	339 (60.3%)	55.6 (47.2 to 63.9)	0.054	−0.084	0.047
One	162 (28.8%)	50.0 (44.4 to 61.1)			
≥ 2	61 (10.9%)	55.6 (44.4 to 61.1)			
Current body weight					
Severely underweight	3 (0.5)	44.4 (43.1 to 51.4)	<0.001	−0.196	<0.001
Underweight	11 (2.0)	52.8 (45.8 to 61.1)			
Normal weight	249 (44.3)	55.6 (47.2 to 63.9)			
Overweight	282 (50.2)	50.0 (44.4 to 58.3)			
Severely obese	17 (3.0)	44.4 (36.1 to 50.0)			
Teaching experience (years)					
< 5	74 (13.2)	52.8 (44.4 to 61.1)	0.737	0.054	0.205
5 to 9	34 (6.0)	50.0 (47.2 to 55.6)			
10 to 14	108 (19.2)	52.8 (44.4 to 61.1)			
15 to 20	69 (12.3)	52.8 (44.4 to 61.1)			
> 20	277 (49.3)	52.8 (47.2 to 63.9)			
Overall health rating					
Poor	20 (3.6)	44.4 (38.2 to 55.6)	<0.001	0.358	<0.001
Fair	122 (21.7)	50.0 (41.7 to 55.6)			
Good	195 (34.7)	50.0 (44.4 to 58.3)			
Very good	154 (27.4)	58.3 (50.0 to 63.9)			
Excellent	71 (12.6)	61.1 (52.8 to 66.7)			

Poor mental well-being (WHO-5 ≤ 50) was reported by 328 (58.4%) participants. Good mental well-being (WHO-5 > 50) was reported by 234 (41.6%). Overall, 137 (24.4%) scored ≤28. No moderate-intensity physical activity was reported by 238 (42.3%) teachers. Daily fruit and vegetable consumption was reported by 168 (29.9%), while 295 (52.5%) consumed unhealthy foods several times per week or almost daily. Poor or very poor sleep quality was reported by 109 (19.4%). Sleep duration was six hours or less for 434 of 561 respondents (77.4%). Tobacco use was reported by 19 (3.4%). Toothbrushing less than twice daily was reported by 157 (27.9%). More than half had last visited a dentist over two years earlier (293; 52.1%), and 348 (61.9%) reported tooth or gum pain at least sometimes during the preceding three months (Supplementary Table S1).

The median lifestyle index score was 52.8 (IQR: 45.1–61.1). Lifestyle scores were positively correlated with self-rated health (*r* = 0.358, *p* < 0.001) and age (*r* = 0.111, *p* = 0.008), and inversely correlated with body weight category (*r* = −0.196, *p* < 0.001) and number of chronic diseases (*r* = −0.084, *p* = 0.047). Scores also differed by place of residence (Kruskal-Wallis *p* = 0.007; Table 2).

The median WHO-5 score was 44.0 (IQR: 32.0–60.0). Well-being was positively correlated with self-rated health (*r* = 0.443, *p* < 0.001; Figure 1) and inversely correlated with the number of chronic diseases (*r* = −0.141, *p* < 0.001). Well-being differed across body weight categories (Kruskal-Wallis *p* = 0.034), although the ordinal correlation was not significant (*r* = −0.080, *p* = 0.058). Other sociodemographic and work characteristics were not significantly associated with well-being (Table 3).

**Figure 1:**
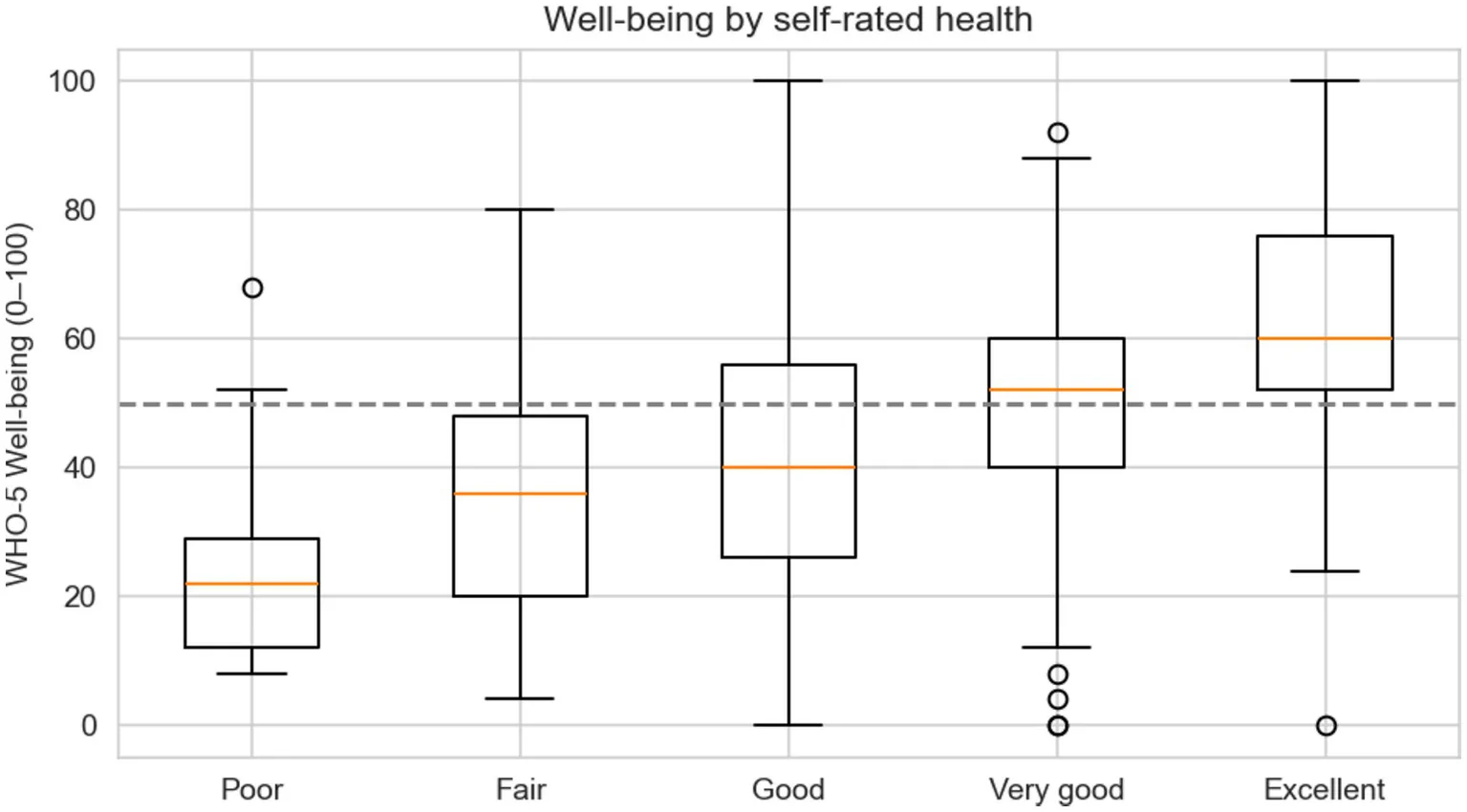
The well-being scores according to overall health ratings.

**Table 3:** Sociodemographic and clinical characteristics of school teachers and their association with mental well-being scores (WHO-5).

Characteristic	Well-being score, Median (IQR)	Kruskal-Wallis *p*	Spearman correlation	Spearman *p*
Overall	44.0 (32.0 to 60.0)			
Age (years)				
20 to 29	56.0 (24.0 to 80.0)	0.679	0.012	0.782
30 to 39	46.0 (28.0 to 60.0)			
40 to 49	44.0 (28.0 to 60.0)			
50 to 59	44.0 (32.0 to 60.0)			
≥ 60	48.0 (40.0 to 55.0)			
Marital status				
Single	40.0 (24.0 to 56.0)	0.200		
Married	48.0 (32.0 to 60.0)			
Widowed	42.0 (36.0 to 56.0)			
Divorced	48.0 (28.0 to 60.0)			
Highest educational level				
High school or less	40.0 (26.0 to 62.0)	0.814	−0.038	0.372
Diploma	52.0 (32.0 to 60.0)			
Bachelor	44.0 (32.0 to 60.0)			
Postgraduate studies	36.0 (24.0 to 60.0)			
Current teaching level				
Primary school	46.0 (32.0 to 60.0)	0.905	0.013	0.764
Intermediate school	44.0 (32.0 to 60.0)			
High school	44.0 (32.0 to 60.0)			
Place of residence				
AlAhsa	28.0 (28.0 to 40.0)	0.286		
AlKhafji	50.0 (38.0 to 60.0)			
Dammam	44.0 (28.0 to 60.0)			
Hafr Al Baten	66.0 (52.0 to 77.0)			
Jubail	44.0 (33.0 to 63.0)			
Khobar	44.0 (36.0 to 60.0)			
Nairiya	46.0 (28.0 to 57.0)			
Qarya AlUlia	48.0 (48.0 to 48.0)			
Qatif	44.0 (32.0 to 60.0)			
Ras Tanura	56.0 (56.0 to 56.0)			
School location				
AlKhafji	56.0 (42.0 to 60.0)	0.281		
Dammam	46.0 (31.0 to 60.0)			
Hafr Al Baten	66.0 (52.0 to 77.0)			
Jubail	44.0 (30.0 to 60.0)			
Khobar	44.0 (36.0 to 60.0)			
Nairiya	44.0 (28.0 to 56.0)			
Qarya AlUlia	32.0 (32.0 to 32.0)			
Qatif	44.0 (28.0 to 56.0)			
Ras Tanura	54.0 (42.0 to 60.0)			
Teaching load (classes per week)				
< 20 classes	44.0 (28.0 to 60.0)	0.636	0.030	0.476
20 to 24 classes	44.0 (32.0 to 60.0)			
> 24 classes	48.0 (36.0 to 60.0)			
Chronic diseases				
None	48.0 (32.0 to 60.0)	0.004	−0.141	<0.001
One	44.0 (28.0 to 56.0)			
≥ 2	40.0 (28.0 to 52.0)			
Current body weight				
Severely underweight	16.0 (12.0 to 36.0)	0.034	−0.080	0.058
Underweight	44.0 (28.0 to 50.0)			
Normal weight	48.0 (36.0 to 60.0)			
Overweight	44.0 (28.0 to 60.0)			
Severely obese	36.0 (16.0 to 52.0)			
Teaching experience (years)				
< 5	48.0 (32.0 to 60.0)	0.337	0.008	0.849
5 to 9	46.0 (24.0 to 60.0)			
10 to 14	44.0 (28.0 to 56.0)			
15 to 20	48.0 (36.0 to 60.0)			
> 20	44.0 (32.0 to 60.0)			
Overall health rating				
Poor	22.0 (12.0 to 29.0)	<0.001	0.443	<0.001
Fair	36.0 (20.0 to 48.0)			
Good	40.0 (26.0 to 56.0)			
Very good	52.0 (40.0 to 60.0)			
Excellent	60.0 (52.0 to 76.0)			

Well-being scores are standardized to a 0–100 scale; higher scores indicate better mental well-being. IQR, interquartile range.

The median satisfaction score was 67.5 (IQR: 52.5–80.0; Figure 2). Teachers were satisfied or very satisfied with their current job (395; 70.3%), work-life balance (260; 46.3%), and work environment (264; 47.0%; Supplementary Table S3). Satisfaction was positively associated with well-being (*p* < 0.001; Figure 3) and self-rated health (*r* = 0.377, *p* < 0.001; Figure 4). Higher weekly teaching load was weakly associated with lower satisfaction (*r* = −0.098, *p* = 0.020). Satisfaction also showed inverse correlations with the number of chronic diseases (*r* = −0.169, *p* < 0.001), body weight category (*r* = −0.096, *p* = 0.023), and educational attainment (*r* = −0.102, *p* = 0.016). Scores differed by residence (*p* = 0.004) and school location (*p* < 0.001; Table 4).

**Figure 2:**
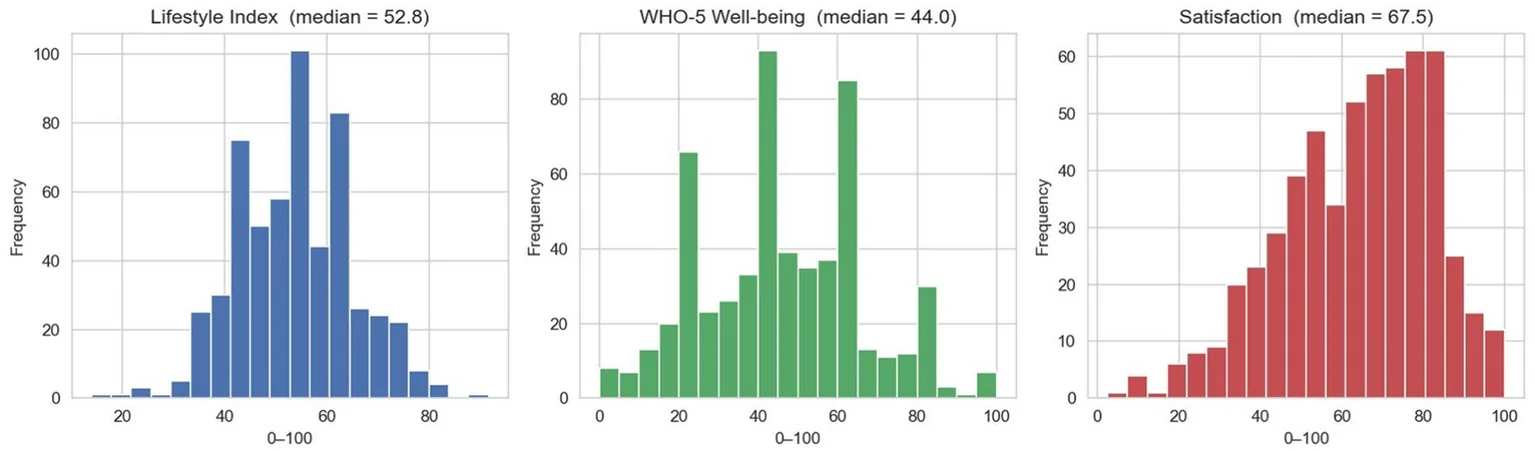
Histograms depicting the distributions of lifestyle index, mental well-being (WHO-5), and satisfaction scores among school teachers.

**Figure 3:**
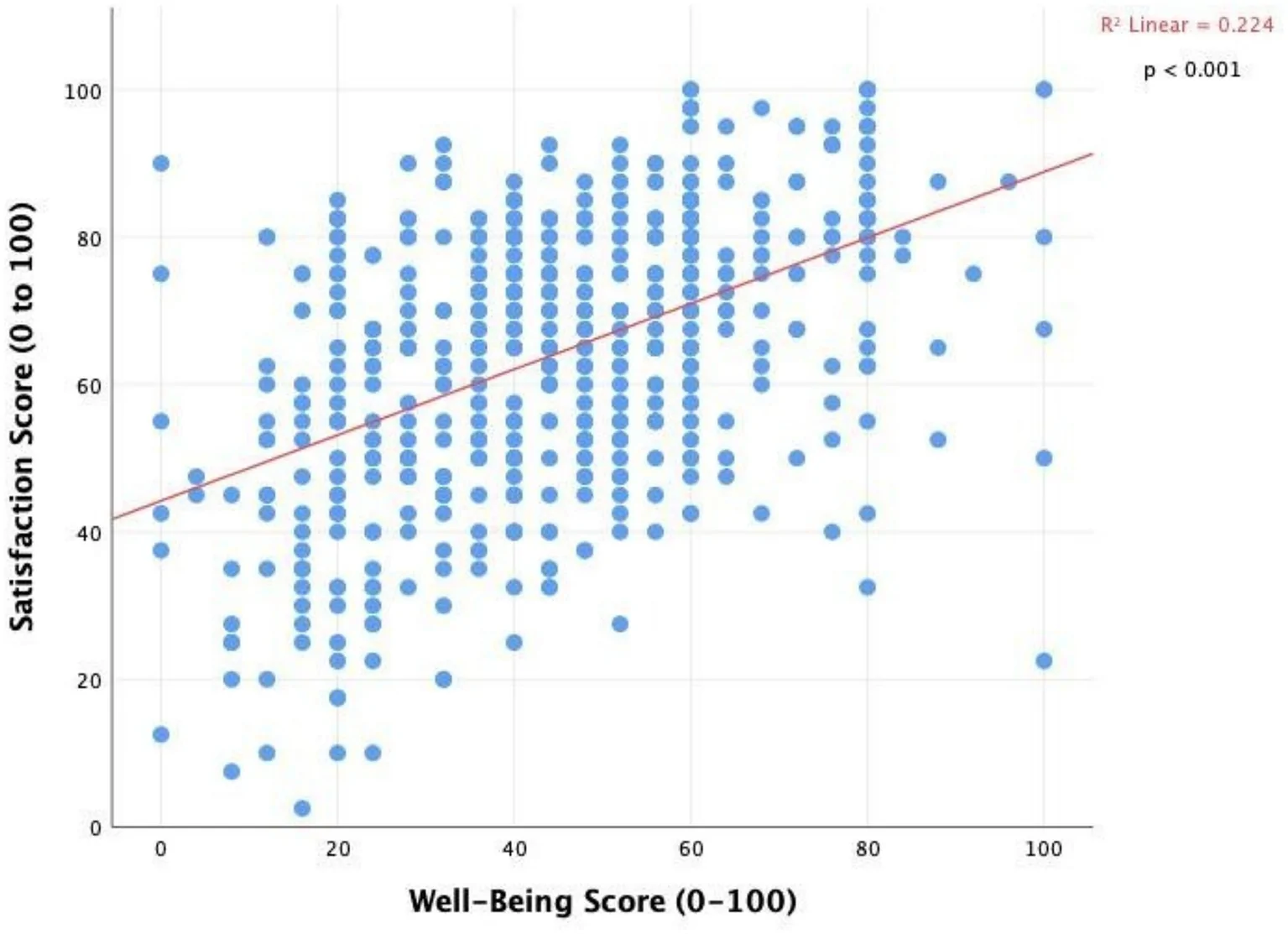
Scatter plot of the correlation between mental well-being score and satisfaction score.

**Figure 4:**
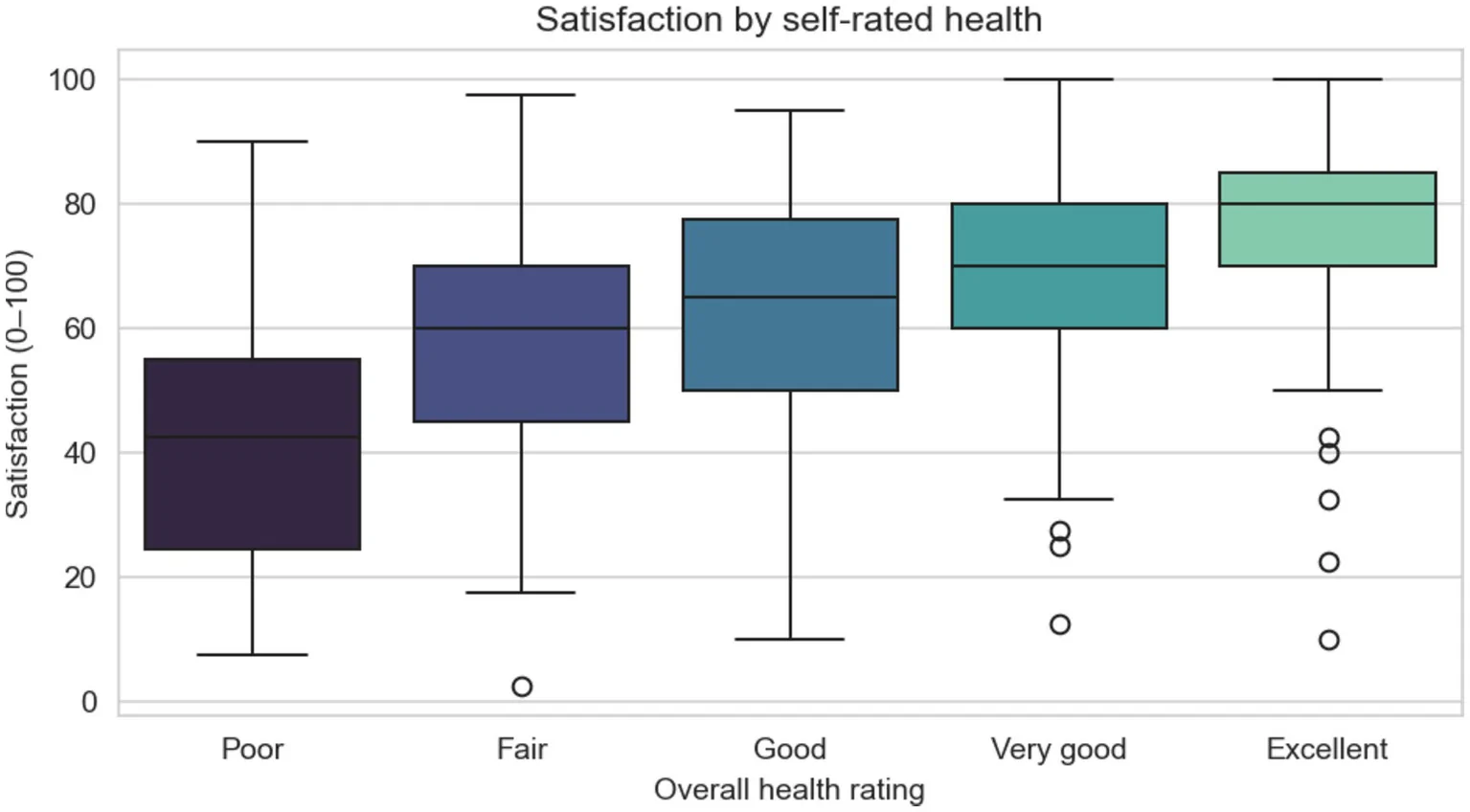
The satisfaction score across overall health ratings.

**Table 4:** Sociodemographic and clinical characteristics of school teachers and their association with satisfaction scores.

Characteristic	Satisfaction score, Median (IQR)	Kruskal-Wallis *p*	Spearman correlation	Spearman *p*
Overall	67.5 (52.5 to 80.0)			
Age (years)				
20 to 29	70.0 (32.5 to 80.0)	0.349	0.069	0.101
30 to 39	65.0 (50.0 to 77.5)			
40 to 49	65.0 (52.5 to 80.0)			
50 to 59	70.0 (55.0 to 80.0)			
≥ 60	65.0 (50.0 to 76.9)			
Marital status				
Single	62.5 (45.0 to 77.5)	0.430		
Married	67.5 (52.5 to 80.0)			
Widowed	65.0 (50.0 to 80.0)			
Divorced	70.0 (52.5 to 80.0)			
Highest educational level				
High school or less	70.0 (58.8 to 81.2)	0.086	−0.102	0.016
Diploma	77.5 (65.0 to 82.5)			
Bachelor	67.5 (52.5 to 80.0)			
Postgraduate studies	62.5 (42.5 to 75.0)			
Current teaching level				
Primary school	67.5 (52.5 to 80.0)	0.354	−0.026	0.540
Intermediate school	67.5 (52.5 to 80.0)			
High school	65.0 (50.0 to 77.5)			
Place of residence				
AlAhsa	75.0 (67.5 to 75.0)	0.004		
AlKhafji	67.5 (53.8 to 85.6)			
Dammam	65.0 (52.5 to 77.5)			
Hafr Al Baten	57.5 (50.0 to 80.6)			
Jubail	75.0 (60.0 to 85.0)			
Khobar	75.0 (56.2 to 84.4)			
Nairiya	71.2 (63.1 to 73.1)			
Qarya AlUlia	87.5 (87.5 to 87.5)			
Qatif	65.0 (50.0 to 77.5)			
Ras Tanura	65.0 (65.0 to 65.0)			
School location				
AlKhafji	75.0 (55.0 to 86.2)	<0.001		
Dammam	67.5 (52.5 to 80.0)			
Hafr Al Baten	57.5 (50.0 to 80.6)			
Jubail	75.0 (61.2 to 85.0)			
Khobar	75.0 (56.2 to 84.4)			
Nairiya	72.5 (70.0 to 77.5)			
Qarya AlUlia	45.0 (45.0 to 45.0)			
Qatif	62.5 (47.5 to 75.0)			
Ras Tanura	73.8 (66.9 to 79.4)			
Teaching load (classes per week)				
< 20 classes	70.0 (55.0 to 81.9)	0.066	−0.098	0.020
20 to 24 classes	65.0 (50.0 to 77.5)			
> 24 classes	67.5 (32.5 to 80.0)			
Chronic diseases				
None	70.0 (55.0 to 80.0)	<0.001	−0.169	<0.001
One	63.8 (48.1 to 77.5)			
≥ 2	57.5 (45.0 to 75.0)			
Current body weight				
Severely underweight	42.5 (38.8 to 62.5)	0.016	−0.096	0.023
Underweight	62.5 (50.0 to 63.8)			
Normal weight	70.0 (55.0 to 82.5)			
Overweight	65.0 (47.5 to 77.5)			
Severely obese	60.0 (42.5 to 80.0)			
Teaching experience (years)				
< 5	65.0 (52.5 to 75.0)	0.541	0.043	0.311
5 to 9	67.5 (43.1 to 80.0)			
10 to 14	66.2 (52.5 to 78.1)			
15 to 20	70.0 (52.5 to 85.0)			
> 20	67.5 (52.5 to 80.0)			
Overall health rating				
Poor	42.5 (24.4 to 55.0)	<0.001	0.377	<0.001
Fair	60.0 (45.0 to 70.0)			
Good	65.0 (50.0 to 77.5)			
Very good	70.0 (60.0 to 80.0)			
Excellent	80.0 (70.0 to 85.0)			

Lifestyle index scores were positively associated with well-being (*p* < 0.001; Figures 5, 6). Sleep quality had a moderate positive correlation with well-being, the largest observed among the lifestyle items (*r* = 0.390, *p* < 0.001; Figure 7). Longer sleep duration was also associated with better well-being (*r* = 0.302, *p* < 0.001). Physical activity frequency (*r* = 0.218), duration (*r* = 0.184), and self-rated activity (*r* = 0.228) were positively correlated with well-being (all *p* < 0.001); activity frequency is shown in Figure 8. Fruit and vegetable consumption was positively correlated with well-being (*r* = 0.179, *p* < 0.001), while unhealthy food consumption was inversely correlated (*r* = −0.118, *p* = 0.005). Toothbrushing frequency showed a positive correlation (*r* = 0.127, *p* = 0.003), and tooth or gum pain showed an inverse correlation (*r* = −0.149, *p* < 0.001). Well-being differed across tobacco-use categories (Kruskal-Wallis *p* = 0.016), but the ordinal correlation was not significant (*p* = 0.064). With only 19 tobacco users, the tobacco analyses are exploratory and should be interpreted cautiously. Time since the last dental visit was not associated with well-being (Table 5). Figures 9, 10 present body weight by unhealthy food consumption and composite scores by self-rated health, respectively.

**Figure 5:**
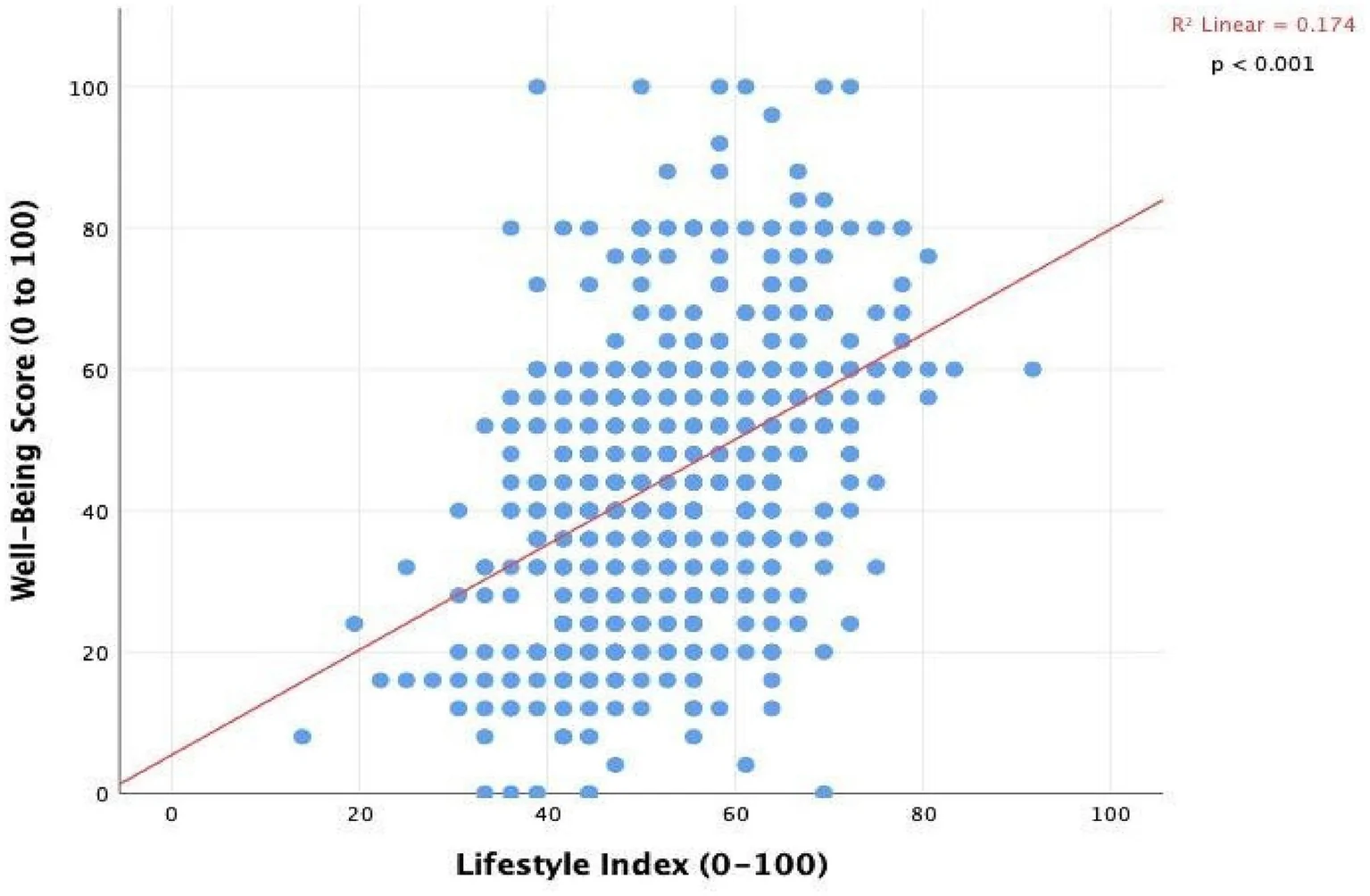
Scatter plot of the correlation between lifestyle index score and mental well-being score.

**Figure 6:**
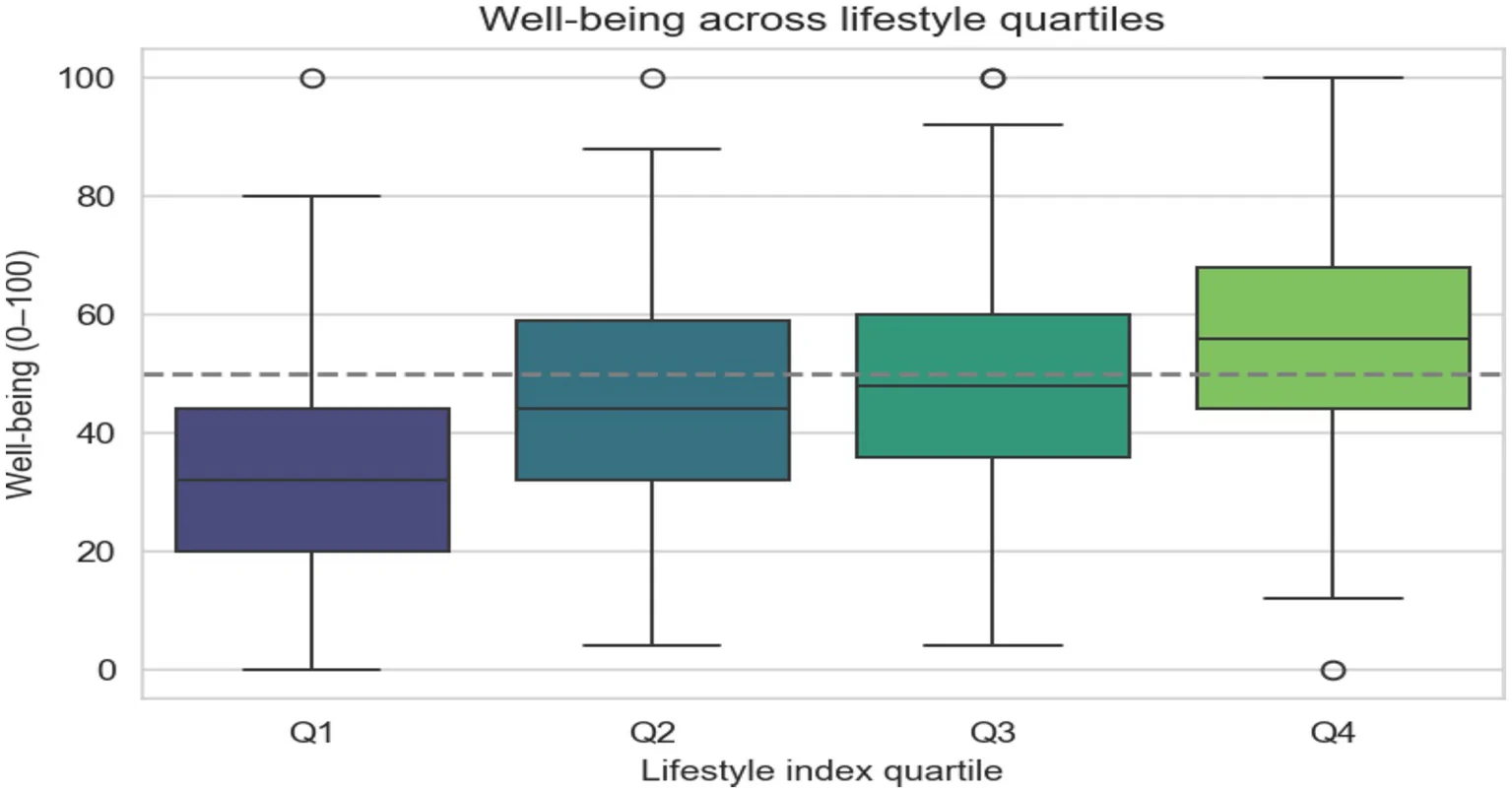
The well-being score across lifestyle index quartiles.

**Figure 7:**
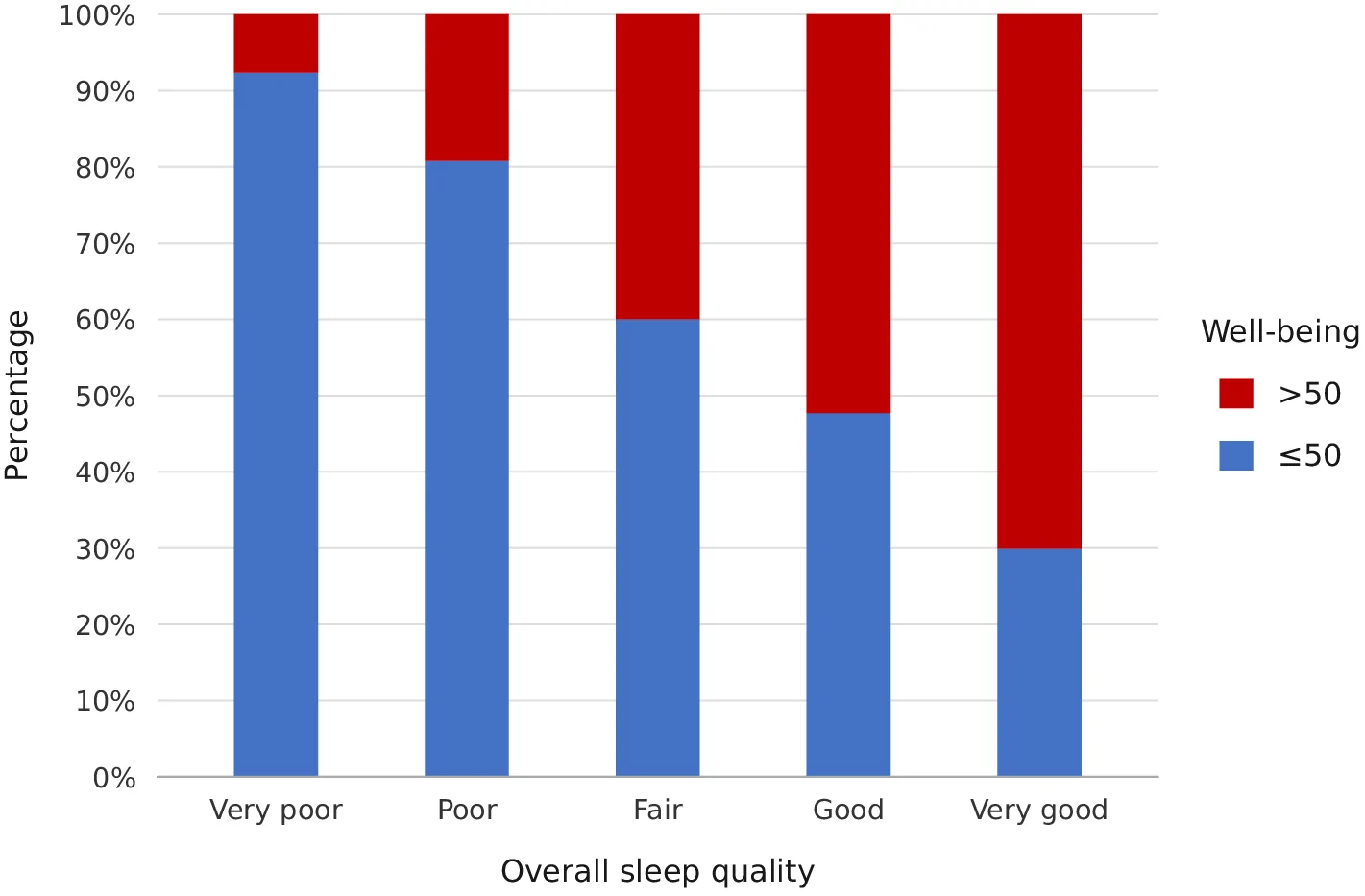
Mental well-being categories by self-rated sleep quality.

**Figure 8:**
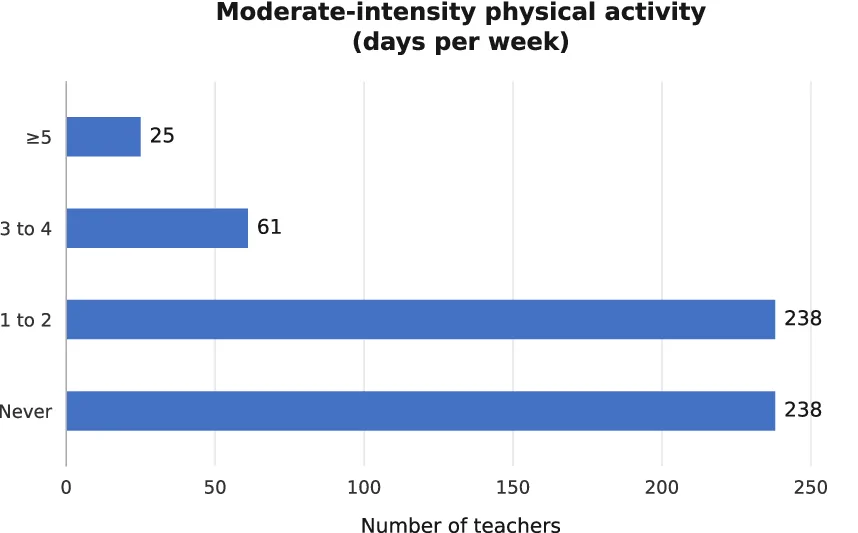
Frequency distribution of moderate-intensity physical activity (days per week) among school teachers.

**Table 5:** Associations between individual lifestyle behavior items and mental well-being scores.

	Well-being, Median (IQR)	Kruskal-Wallis *p*	Spearman correlation	Spearman *p*
Overall health rating				
Poor	22.0 (12.0 to 29.0)	<0.001	0.443	<0.001
Fair	36.0 (20.0 to 48.0)			
Good	40.0 (26.0 to 56.0)			
Very good	52.0 (40.0 to 60.0)			
Excellent	60.0 (52.0 to 76.0)			
Moderate-intensity physical activity (days per week)				
Never	40.0 (24.0 to 56.0)	<0.001	0.218	<0.001
1 to 2	44.0 (32.0 to 60.0)			
3 to 4	56.0 (40.0 to 68.0)			
≥ 5	60.0 (48.0 to 60.0)			
Physical activity duration (minutes)				
Less than 15	40.0 (24.0 to 56.0)	<0.001	0.184	<0.001
15 to 29	48.0 (37.0 to 60.0)			
30 to 59	52.0 (36.0 to 60.0)			
≥ 60	60.0 (32.0 to 68.0)			
Overall physical activity level				
Not active at all	28.0 (15.0 to 44.0)	<0.001	0.228	<0.001
Not very active	40.0 (24.0 to 56.0)			
Moderately active	44.0 (32.0 to 60.0)			
Active	56.0 (40.0 to 62.0)			
Very active	46.0 (24.0 to 62.0)			
Fruit and vegetable consumption (times per week)				
Rarely / Never	38.0 (24.0 to 51.0)	<0.001	0.179	<0.001
1 to 2	44.0 (28.0 to 60.0)			
3 to 4	48.0 (35.0 to 60.0)			
Daily	50.0 (36.0 to 60.0)			
Unhealthy food consumption				
Rarely / Never	52.0 (32.0 to 60.0)	<0.001	−0.118	0.005
Once a week or less	46.0 (32.0 to 60.0)			
Several times per week	48.0 (36.0 to 60.0)			
Almost daily	36.0 (20.0 to 50.0)			
Overall sleep quality over the past month				
Very poor	18.0 (12.0 to 35.0)	<0.001	0.390	<0.001
Poor	28.0 (20.0 to 44.0)			
Fair	44.0 (32.0 to 60.0)			
Good	52.0 (40.0 to 60.0)			
Very good	60.0 (44.0 to 72.0)			
Average sleep per night (hours)				
< 5	36.0 (20.0 to 48.0)	<0.001	0.302	<0.001
5 to 6	44.0 (32.0 to 60.0)			
7 to 8	56.0 (40.0 to 64.0)			
> 8	60.0 (40.0 to 60.0)			
Current tobacco use				
No	44.0 (32.0 to 60.0)	0.016	−0.078	0.064
Yes, occasionally	28.0 (21.0 to 39.0)			
Yes, daily	48.0 (44.0 to 80.0)			
Daily tobacco use (frequency)				
< 5	36.0 (23.0 to 50.0)	0.357	−0.197	0.420
5 to 10	42.0 (27.0 to 61.0)			
> 10	28.0 (14.0 to 28.0)			
Daily toothbrushing frequency				
0	36.0 (24.0 to 46.0)	0.016	0.127	0.003
1	44.0 (28.0 to 56.0)			
2	48.0 (31.0 to 60.0)			
> 2	48.0 (36.0 to 60.0)			
Last dental visit				
Never	50.0 (23.0 to 69.0)	0.322	0.039	0.356
Within past 6 months	40.0 (24.0 to 56.0)			
About a year	44.0 (32.0 to 60.0)			
More than 2 years	48.0 (32.0 to 60.0)			
Tooth or gum pain in the past 3 months				
Never	48.0 (36.0 to 60.0)	0.003	−0.149	<0.001
Rarely	50.0 (40.0 to 60.0)			
Sometimes	44.0 (28.0 to 59.0)			
Often	28.0 (24.0 to 38.0)			
Most of the time	40.0 (24.0 to 52.0)			
Always	40.0 (29.0 to 56.0)			

**Figure 9:**
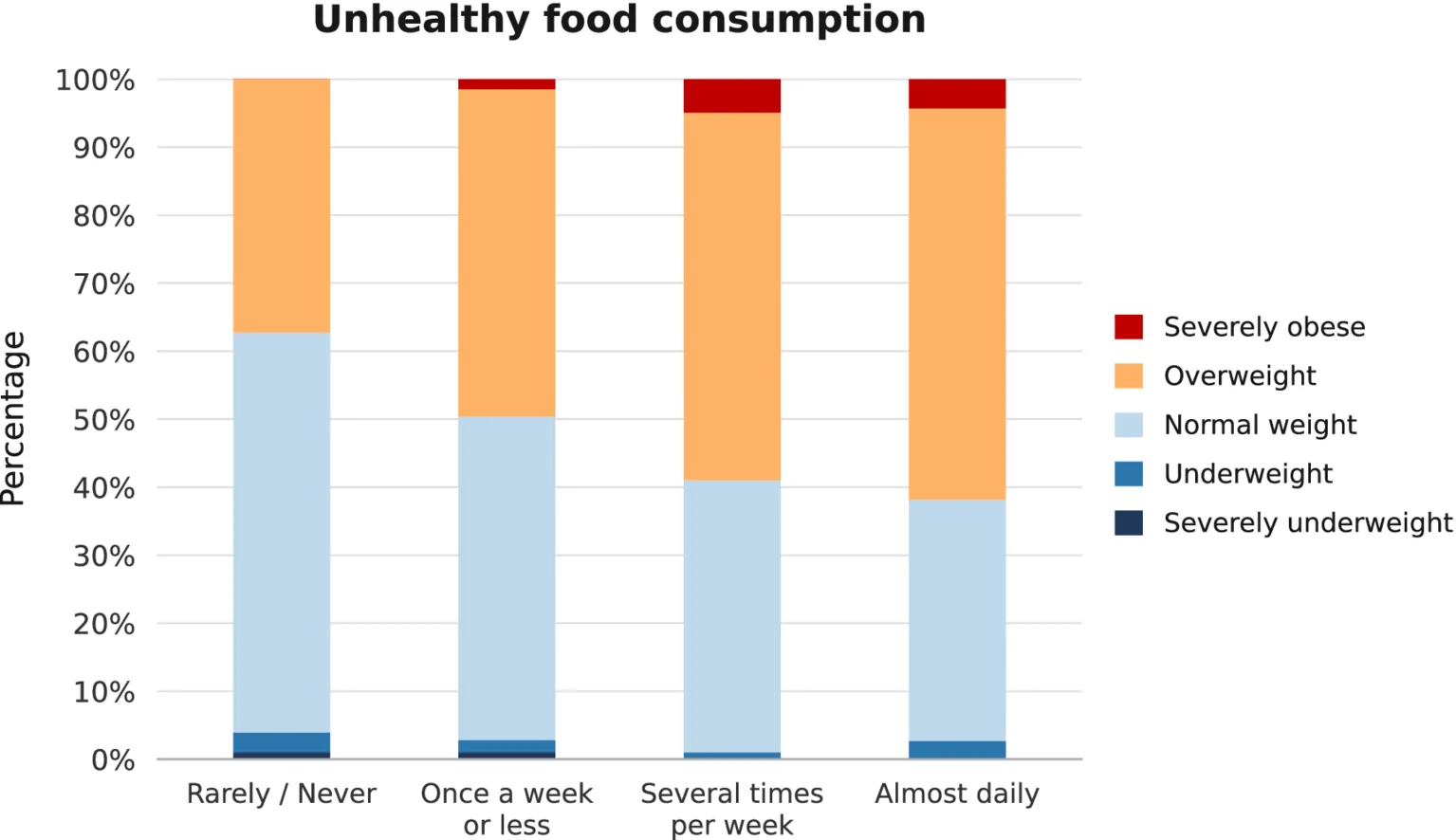
Body weight distribution by frequency of unhealthy food consumption.

**Figure 10:**
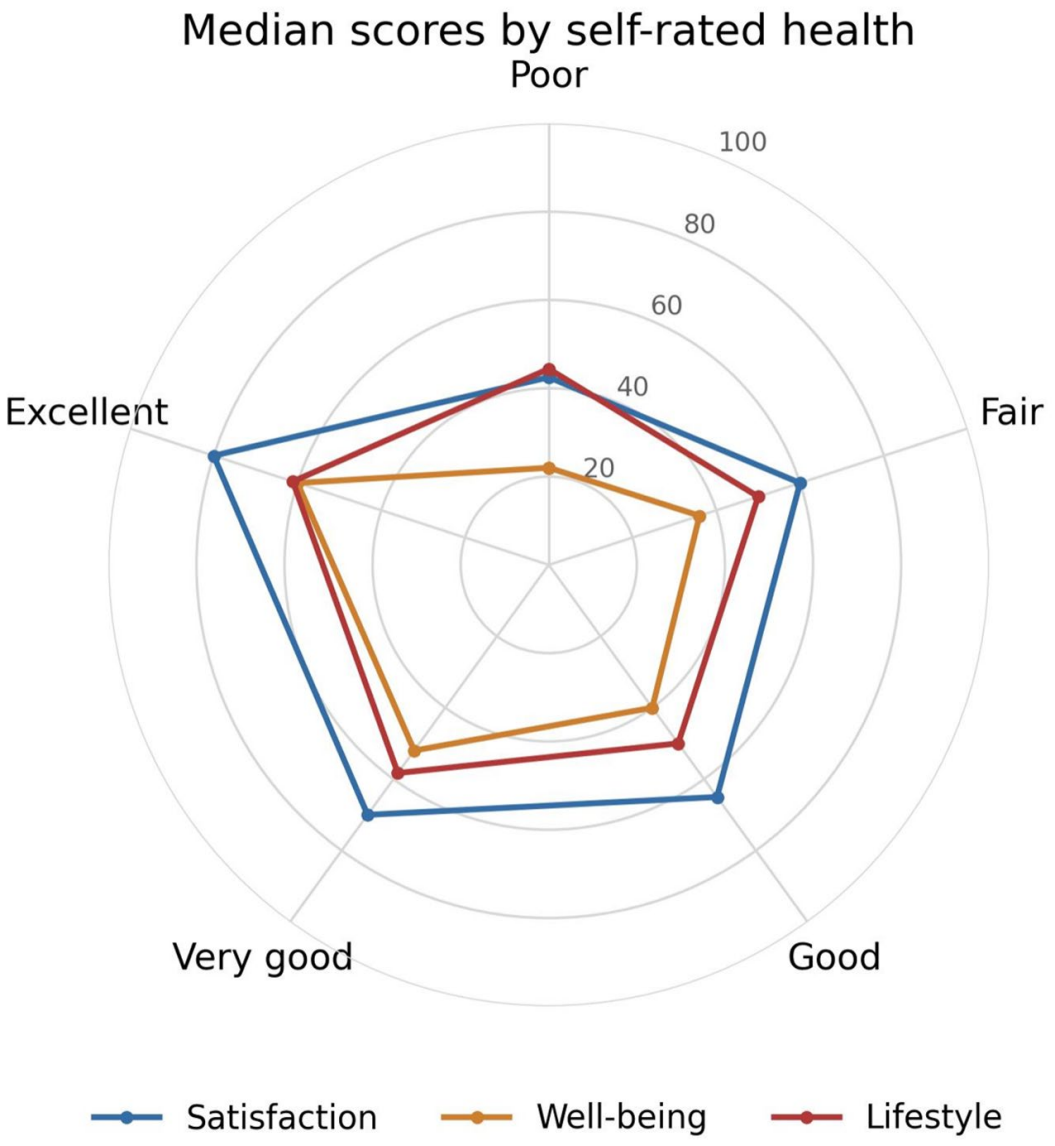
Radar plot of median lifestyle index, mental well-being, and satisfaction scores by overall self-rated health.

Lifestyle index score, satisfaction, and self-rated health remained associated with good mental well-being after adjustment (Table 1). Each one-point increase in lifestyle score (OR = 1.051, 95% CI: 1.030–1.073) and satisfaction score (OR = 1.035, 95% CI: 1.022–1.048) was associated with higher odds of good well-being (both *p* < 0.001). Teachers reporting excellent rather than poor health also had higher odds (OR = 7.038, 95% CI: 1.302–38.031, *p* = 0.023). The number of chronic diseases was not significant after adjustment (overall *p* = 0.679).

### 3.1 Associations of lifestyle behaviors and mental well-being with absenteeism

At least one day of absence per year due to physical health problems was reported by 478 (85.1%) teachers, and absence due to mental health problems by 225 (40.0%). Five or more days were reported by 167 (29.7%) and 46 (8.2%), respectively (Table 6). Lifestyle scores were inversely correlated with oral health-related absence (*r* = −0.246), physical health-related absence (*r* = −0.171), and mental health-related absence (*r* = −0.148; all *p* < 0.001; Table 6). Mental health-related absence had the largest observed inverse correlation with well-being among the absence measures, although it was weak (*r* = −0.175, *p* < 0.001). Smaller inverse correlations were found for physical health-related absence (*r* = −0.104, *p* = 0.014) and oral health-related absence (*r* = −0.097, *p* = 0.022; Table 7).

**Table 6:** Frequency of work absence and its association with lifestyle index scores.

	*N* (%)	Lifestyle index, Median (IQR)	Kruskal-Wallis *p*	Spearman correlation	Spearman *p*
Work absence due to dental pain/oral health problems					
Never	278 (49.5)	55.6 (47.2 to 63.9)	<0.001	−0.246	<0.001
Rarely	151 (26.9)	52.8 (47.2 to 63.9)			
Sometimes	116 (20.6)	50.0 (41.7 to 56.2)			
Often	4 (0.7)	41.7 (41.7 to 45.1)			
Most of the time	11 (2.0)	41.7 (38.9 to 47.2)			
Always	2 (0.4)	38.9 (37.5 to 40.3)			
Physical health-related absence (days/year)					
0	84 (14.9)	55.6 (47.2 to 63.9)	<0.001	−0.171	<0.001
1 to 2	162 (28.8)	55.6 (47.2 to 63.9)			
3 to 4	149 (26.5)	52.8 (47.2 to 63.9)			
≥ 5	167 (29.7)	50.0 (44.4 to 58.3)			
Mental health-related absence (days/year)					
0	337 (60.0)	55.6 (47.2 to 63.9)	<0.001	−0.148	<0.001
1 to 2	127 (22.6)	55.6 (47.2 to 63.9)			
3 to 4	52 (9.3)	50.0 (43.8 to 55.6)			
≥ 5	46 (8.2)	47.2 (41.7 to 55.6)			

**Table 7:** Frequency of work absence and its association with mental well-being scores.

	Well-being, Median (IQR)	Kruskal-Wallis *p*	Spearman correlation	Spearman *p*
Work absence due to dental pain/oral health problems				
Never	48.0 (32.0 to 60.0)	0.005	−0.097	0.022
Rarely	48.0 (36.0 to 60.0)			
Sometimes	40.0 (24.0 to 56.0)			
Often	24.0 (24.0 to 25.0)			
Most of the time	32.0 (22.0 to 40.0)			
Always	52.0 (52.0 to 52.0)			
Physical health-related absence (days/year)				
0	44.0 (27.0 to 60.0)	0.004	−0.104	0.014
1 to 2	48.0 (36.0 to 60.0)			
3 to 4	48.0 (32.0 to 60.0)			
≥ 5	40.0 (24.0 to 56.0)			
Mental health-related absence (days/year)				
0	48.0 (36.0 to 60.0)	<0.001	−0.175	<0.001
1 to 2	40.0 (30.0 to 56.0)			
3 to 4	42.0 (24.0 to 52.0)			
≥ 5	34.0 (24.0 to 48.0)			

### 3.2 Qualitative findings

Five themes described teachers’ experiences of work, daily routines, and health (Table 8; Figure 11). The burden of the workday and work carried into the home were linked through accounts of limited control over time and reduced opportunities for recovery. Barriers to a healthy lifestyle reflected competing work and family demands. One teacher explained, “I started getting home late, and I have a child, which made things exhausting, so I stopped going to the gym.”

**Table 8:** Themes and subthemes describing female teachers’ experiences of workload, lifestyle, and health.

Theme	Subthemes	Summary of findings
The burden of the workday	Heavy teaching loads; extended on-site hours; administrative documentation; Madrasati digital learning platform requirements; unplanned substitution classes	Teachers described demanding and fragmented workdays. Teaching loads ranged from 18 to 24 classes per week. Teachers reported that attendance requirements recorded through HUDURI, the Ministry of Education attendance application, kept them on site beyond teaching hours. Documentation tasks and unplanned substitution classes added to their workload.
Barriers to a healthy lifestyle	Fatigue; family responsibilities; limited physical activity; irregular eating; reduced sleep	Fatigue after school and family responsibilities limited regular physical activity. Teachers described skipping breakfast, relying on coffee and sweets during the day, and reduced or poor sleep as lesson preparation and digital-platform tasks continued into the evening.
Emotional and cognitive demands	Large classes; challenging parent interactions; limited administrative recognition; end-of-week exhaustion; coping strategies	Large classes, difficult interactions with parents, and limited recognition from school administration were described as persistent emotional demands. Several teachers used the term *burnout* to describe cumulative exhaustion, particularly by the end of the working week. Coping strategies included rest, leisure activities, religious practice, physical activity, and support from colleagues.
Work carried into the home	Lesson preparation; marking; platform-related tasks; quality assurance documentation	Work often continued after school through lesson preparation, marking, and platform-related tasks. This reduced time for family, social relationships, and recovery. A smaller number of teachers described clearer work-home boundaries that helped protect their personal time.
Occupational health concerns	Voice and throat complaints; musculoskeletal pain; prolonged standing; board writing; perceived worsening of chronic conditions	Teachers reported voice and throat complaints and musculoskeletal pain that they associated with prolonged standing and board writing. Some felt that workload and stress worsened their existing health problems.

**Figure 11:**
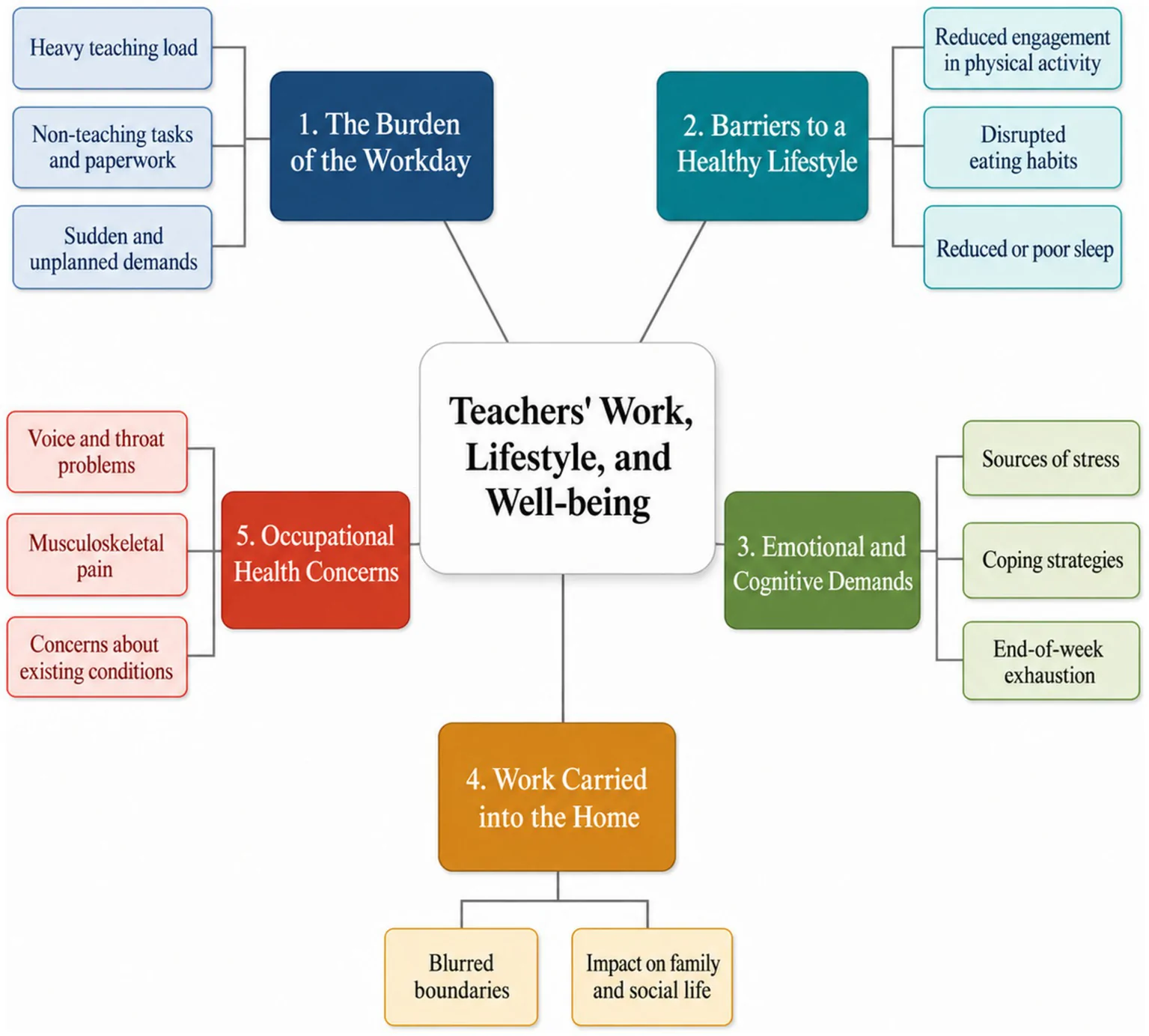
Thematic map of the qualitative findings.

Emotional and cognitive demands included difficult classroom and parent interactions and limited administrative recognition. Teachers described satisfaction and meaning in their work, as well as support from colleagues. Some experienced teachers described managing pressure more effectively, while others needed weekends to recover: “By Thursday when the workweek ends, I don’t feel like going out or doing anything,” one teacher said.

Occupational health concerns included voice complaints and musculoskeletal pain that teachers associated with their work. Some felt that stress worsened existing health conditions. Experiences varied: some maintained exercise, prepared meals at home, or protected personal time. Participants suggested reducing teaching loads and non-teaching documentation and planning the academic year in advance. They also called for better facilities, smaller classes, and stronger administrative and staffing support. They suggested providing healthy food at school, potentially through partnerships with external providers.

### 3.3 Integration of quantitative and qualitative findings

The interviews described how working conditions shaped teachers’ daily routines and experiences of health. Better sleep quality and longer sleep were associated with better well-being (Table 5). Interviewees described delayed bedtimes and exhaustion as work continued into the evening. Accounts of fatigue, irregular meals, and limited exercise provided context for the positive associations of physical activity and fruit and vegetable consumption with well-being.

Higher satisfaction scores remained associated with good well-being after adjustment, while higher teaching load was weakly associated with lower satisfaction (Tables 1, 4). Teachers described meaningful work and collegial support alongside heavy workload and limited administrative recognition. Lower lifestyle and well-being scores were associated with more health-related absence (Tables 6, 7). One participant described taking time off after continuing to teach while ill.

## 4 Discussion

### 4.1 Principal findings

Teacher health is an important part of school health promotion and population health, with staff quality of life as a goal in its own right. The WSCC framework and WHO and UNESCO standards place staff health within a comprehensive school health approach (32, 34). More than half of participants reported poor mental well-being. Sleep quality showed a moderate positive correlation with well-being. Lifestyle, satisfaction, and self-rated health remained associated with well-being after adjustment. Lower lifestyle and well-being scores were also associated with more health-related absence. Interview accounts described how work demands limited rest and healthy routines. These findings support addressing working conditions alongside individual health behaviors in school health planning.

### 4.2 Lifestyle behaviors

Physical activity was limited: 42.3% of teachers reported no moderate-intensity activity. Similar concerns have been reported among Turkish primary teachers and public-school teachers in Jeddah (12, 42). Interview participants described fatigue, attendance requirements, and family responsibilities as barriers to exercise. Only 29.9% reported daily fruit and vegetable consumption. Interview accounts of skipped or hurried breakfasts and reliance on coffee and sweets suggest that regular meal breaks and access to healthy food at school deserve attention. Lower fruit intake has also been associated with emotional exhaustion among Flemish teachers (16). Tobacco use was uncommon (3.4%). Earlier studies in Syria and Bahrain reported lower smoking prevalence among female teachers than male teachers (18, 19). More than half had not visited a dentist for over two years. Saudi studies have reported fair conceptual knowledge of oral health (21) and adequate oral health knowledge among teachers (20). School health promotion should combine health education with practical access to healthy food, physical activity, and preventive dental care.

Salary, food costs, healthy food availability at school, and time for shopping were not assessed systematically. Their contribution to dietary patterns therefore remains uncertain. Future studies should examine these factors alongside meal breaks. School food partnerships should consider affordability as well as availability.

### 4.3 Mental well-being

Poor mental well-being was common. Depressive symptoms have been reported among teachers in Dammam, Khobar, and Qatif (5). Australian teachers also reported greater psychological distress than other professionals (43). Participants described heavy teaching loads, documentation, unplanned demands, and exhaustion by the end of the week. These accounts are consistent with associations between work burden, limited support, and psychological distress among teachers in Jeddah (6). Interviews also described faith, peer support, and professional meaning as sources of support. In a cross-sectional study, better teacher well-being was associated with better student well-being (33).

The female-only sample is relevant to the work-family demands described in the interviews. Some participants reported childcare and household responsibilities that reduced time for exercise and recovery. These accounts provide context for the findings but do not establish differences between female and male teachers. Comparative studies are needed to examine whether workplace conditions and family demands differ by sex.

### 4.4 Association between lifestyle behaviors and mental well-being

Sleep quality showed a moderate positive correlation with well-being (*r* = 0.390), the largest observed among the lifestyle items. This is consistent with research linking sleep quality with psychological health in teaching populations (15, 22). Interview participants described lesson preparation and digital-platform tasks reducing their time for rest. Physical activity and fruit and vegetable consumption showed smaller positive correlations with well-being. The physical activity finding is consistent with evidence from teachers in Al-Madinah (44). Prospective research has also linked healthier lifestyles with a lower likelihood of depressive symptoms (23). These findings support protecting time for rest within workplace health promotion.

The adjusted lifestyle association is easier to interpret over a 10-point difference on the 0–100 scale. This corresponds to an approximate OR of 1.64 (95% CI: 1.34–2.02), calculated from the reported per-point estimates. This is an association with the odds of good well-being, not an increase in its probability or evidence of treatment benefit. A clinically meaningful difference in this composite has not been established.

### 4.5 Satisfaction, absenteeism, and workplace health promotion

Satisfaction was associated with good mental well-being after adjustment. Related Saudi research has reported an association between resilience and job satisfaction among university faculty (28). Among the absence measures, oral health-related absence had the largest observed inverse correlation with lifestyle scores, while mental health-related absence had the largest observed inverse correlation with well-being. Both correlations were weak. Previous Saudi research has also linked sleep disturbance and nervousness with absence due to musculoskeletal pain (45).

Teachers’ accounts of workload, attendance requirements, and administrative demands suggest that workplace health promotion should address organizational conditions alongside individual behaviors. HUDURI is the Ministry of Education’s mobile attendance system, which uses location and biometric verification to record staff attendance (46). Participants described attendance requirements as extending their time at school beyond scheduled teaching. They also reported fatigue and limited recovery time. These accounts concern attendance arrangements alongside teaching duties. The study did not measure required on-site hours or break monitoring and cannot isolate an effect of the application itself. Other research has linked work-related factors with psychosocial health among Australian teachers (2). Management support has also been associated with healthier teacher lifestyles (35). School health promotion should address workload and scheduling. It should also support healthy routines and access to psychosocial and administrative support (3, 47). Sustained investment should prioritize teachers’ health and quality of life, with staff involvement in program design and evaluation.

Intervention studies offer options for school health promotion. Guided sleep support has reduced insomnia among teachers (48). Exercise and behavioral support have improved some activity outcomes, although benefits varied across measures (49). A school program addressing nutrition and physical activity helped prevent weight gain among staff (50). An organizational intervention showed no benefit for recovery or vitality (51). These findings support evaluating staff health programs alongside improvements in working conditions.

Madrasati is the Saudi Ministry of Education’s digital learning platform, used for tasks such as setting and managing student assignments (52). Guided recovery training for teachers has included strategies for ending the workday and separating work from personal time (48). This trial did not evaluate Madrasati or school-wide digital workload rules. Protected time for platform tasks and agreed limits on after-hours requests are practical options for future evaluation in Saudi schools.

### 4.6 Strengths and limitations

The study assessed several lifestyle domains alongside well-being, satisfaction, and absenteeism. It used the WHO-5 and interviews that provided context for the quantitative findings. The cross-sectional design prevents causal inference, and self-reported measures may be affected by recall and social desirability bias. The sample was restricted to female government-school teachers in the Eastern Province, which limits generalizability to other teacher populations.

Absence was recalled for the preceding year and was not verified against school records. The categories such as 0, 1–2, 3–4, and ≥5 days limit assessment of dose–response relationships. The large number of unadjusted tests increases the risk of false-positive findings. Small associations should be judged by their magnitude and uncertainty, not only by *p* values (53). Selection based on univariable *p* values can produce unstable estimates and overfitting (54). The lifestyle index combines behaviors with dental attendance and oral symptoms. Toothbrushing and preventive dental care are relevant to health promotion, and oral health shares risk factors with other health outcomes (55). However, tooth or gum pain is a symptom rather than a behavior. The composite should therefore be interpreted as a broad summary of reported lifestyle and oral health, not a validated single construct. Item-level associations are reported separately in Table 5. The available scoring description does not fully specify category coding for the two composite measures. The handling of incomplete responses in score calculation and regression is also insufficiently documented. These gaps limit reproducibility. Random interview recruitment may have missed less common experiences. The qualitative findings provide a contextual account of this sample and should not be regarded as exhaustive.

## 5 Conclusion

Teachers’ health and quality of life should be priorities in school health promotion and education policy. More than half of the teachers reported poor mental well-being. Unhealthy patterns of physical activity, diet, and sleep were common, alongside oral health concerns. Well-being was associated with sleep, physical activity, dietary behaviors, and oral health. Lifestyle scores, satisfaction, and self-rated health remained associated with good well-being after adjustment. Higher teaching load was weakly associated with lower satisfaction, while lower lifestyle and well-being scores were associated with more health-related absence. Interviews described heavy workload and limited support as barriers to healthy behaviors and well-being. Policymakers should include staff health promotion in school planning. Priorities include manageable work demands, health education, and access to preventive care. Guided sleep support could be adapted in Arabic around local work and family routines. School nutrition partnerships could improve access to affordable healthy meals, consistent with the healthy lifestyle priorities of Saudi Vision 2030 (4). These proposals draw on teacher sleep and school worksite interventions (48, 50). Adaptation elsewhere should reflect local schedules and food practices. Their feasibility and effects on teacher health should be evaluated before wider implementation.

## Data Availability

The raw data supporting the conclusions of this article will be made available by the authors, without undue reservation.
