# Supplementary material for: An investigation of lifestyle behaviors, mental well-being, and work-related outcomes among female teachers: mixed-methods evidence for occupational health promotion
Source: Front Public Health. 2026 Oct 22;14:1993916. doi: 10.3389/fpubh.2026.1993916 (PMC13736735; doi:10.3389/fpubh.2026.1993916)
Supplement: Supplementary file 1 [file Supplementary_file_1.docx]

**Supplementary Material**

**Table S1. Distribution of Responses to Lifestyle Behavior Items**

|  | **N (%)** | **Median (IQR)** |
| --- | --- | --- |
| **Moderate-intensity physical activity (days per week)** |  |  |
| Never | 238 (42.3) | 1.0 (0 to 1.0) |
| 1 to 2 | 238 (42.3) | 1.0 (0 to 1.0) |
| 3 to 4 | 61 (10.9) | 1.0 (0 to 1.0) |
| ≥ 5 | 25 (4.4) | 1.0 (0 to 1.0) |
| **Physical activity duration (minutes)** |  |  |
| Less than 15 | 231 (44.5) | 1.0 (0 to 1.0) |
| 15 to 29 | 174 (33.5) | 1.0 (0 to 1.0) |
| 30 to 59 | 89 (17.1) | 1.0 (0 to 1.0) |
| ≥ 60 | 25 (4.8) | 1.0 (0 to 1.0) |
| **Overall physical activity level** |  |  |
| Not active at all | 24 (4.3) | 2.0 (2.0 to 2.0) |
| Not very active | 97 (17.3) | 2.0 (2.0 to 2.0) |
| Moderately active | 322 (57.3) | 2.0 (2.0 to 2.0) |
| Active | 107 (19.0) | 2.0 (2.0 to 2.0) |
| Very active | 12 (2.1) | 2.0 (2.0 to 2.0) |
| **Fruit and vegetable consumption (times per week)** |  |  |
| Rarely / Never | 62 (11.0) | 2.0 (1.0 to 3.0) |
| 1 to 2 | 200 (35.6) | 2.0 (1.0 to 3.0) |
| 3 to 4 | 132 (23.5) | 2.0 (1.0 to 3.0) |
| Daily | 168 (29.9) | 2.0 (1.0 to 3.0) |
| **Unhealthy food consumption** |  |  |
| Rarely / Never | 75 (13.3) | 2.0 (1.0 to 2.0) |
| Once a week or less | 192 (34.2) | 2.0 (1.0 to 2.0) |
| Several times per week | 224 (39.9) | 2.0 (1.0 to 2.0) |
| Almost daily | 71 (12.6) | 2.0 (1.0 to 2.0) |
| **Overall sleep quality over the past month** |  |  |
| Very poor | 26 (4.6) | 2.0 (2.0 to 3.0) |
| Poor | 83 (14.8) | 2.0 (2.0 to 3.0) |
| Fair | 241 (42.9) | 2.0 (2.0 to 3.0) |
| Good | 159 (28.3) | 2.0 (2.0 to 3.0) |
| Very good | 53 (9.4) | 2.0 (2.0 to 3.0) |
| **Average sleep per night (hours)** |  |  |
| < 5 | 122 (21.7) | 1.0 (1.0 to 1.0) |
| 5 to 6 | 312 (55.6) | 1.0 (1.0 to 1.0) |
| 7 to 8 | 118 (21.0) | 1.0 (1.0 to 1.0) |
| > 8 | 9 (1.6) | 1.0 (1.0 to 1.0) |
| **Current tobacco use** |  |  |
| No | 543 (96.6) | 0 |
| Yes, occasionally | 14 (2.5) | 0 |
| Yes, daily | 5 (0.9) | 0 |
| **Daily tobacco use (frequency)** |  |  |
| < 5 | 12 (63.2) | 0 |
| 5 to 10 | 4 (21.1) | 0 |
| > 10 | 3 (15.8) | 0 |
| **Daily toothbrushing frequency** |  |  |
| 0 | 19 (3.4) | 2.0 (1.0 to 2.0) |
| 1 | 138 (24.6) | 2.0 (1.0 to 2.0) |
| 2 | 304 (54.1) | 2.0 (1.0 to 2.0) |
| > 2 | 101 (18.0) | 2.0 (1.0 to 2.0) |
| **Last dental visit** |  |  |
| Never | 10 (1.8) | 3.0 (2.0 to 3.0) |
| Within past 6 months | 99 (17.6) | 3.0 (2.0 to 3.0) |
| About a year | 160 (28.5) | 3.0 (2.0 to 3.0) |
| More than 2 years | 293 (52.1) | 3.0 (2.0 to 3.0) |
| **Tooth or gum pain in the past 3 months** |  |  |
| Never | 128 (22.8) | 2.0 (1.0 to 2.0) |
| Rarely | 86 (15.3) | 2.0 (1.0 to 2.0) |
| Sometimes | 242 (43.1) | 2.0 (1.0 to 2.0) |
| Often | 11 (2.0) | 2.0 (1.0 to 2.0) |
| Most of the time | 69 (12.3) | 2.0 (1.0 to 2.0) |
| Always | 26 (4.6) | 2.0 (1.0 to 2.0) |

*Note. Values are presented as N (%) for each response category, with the item-level median (IQR) on its recoded ordinal scale shown in the final column. IQR = interquartile range.* Response totals are 561 for sleep duration and 519 for physical activity duration. Tobacco quantity is reported among 19 tobacco users. Other listed items have 562 responses.

**Table S2. Distribution of Responses to the Five WHO-5 Well-Being Items**

|  | **At no time** | **Some of the time** | **Less than half of the time** | **More than half of the time** | **Most of the time** | **All of the time** | **Median (IQR)** |
| --- | --- | --- | --- | --- | --- | --- | --- |
| Felt cheerful and in good spirits | 18 (3.2%) | 99 (17.6%) | 162 (28.8%) | 186 (33.1%) | 83 (14.8%) | 14 (2.5%) | 3.0 (2.0 to 3.0) |
| Felt calm and relaxed | 30 (5.3%) | 110 (19.6%) | 155 (27.6%) | 180 (32%) | 73 (13%) | 14 (2.5%) | 2.0 (2.0 to 3.0) |
| Felt active and vigorous | 31 (5.5%) | 108 (19.2%) | 191 (34%) | 167 (29.7%) | 56 (10%) | 9 (1.6%) | 2.0 (2.0 to 3.0) |
| Felt fresh and rested when you woke up | 54 (9.6%) | 142 (25.3%) | 167 (29.7%) | 129 (23%) | 59 (10.5%) | 11 (2%) | 2.0 (1.0 to 3.0) |
| Daily life filled with things that interest you | 38 (6.8%) | 125 (22.2%) | 163 (29%) | 158 (28.1%) | 64 (11.4%) | 14 (2.5%) | 2.0 (1.0 to 3.0) |

*Note. Values are presented as N (%) for each response category (rated 0 = "At no time" to 5 = "All of the time"), with the item-level median (IQR) shown in the final column.*

**Table S3. Distribution of Responses to the Eight Satisfaction Items**

|  | **N (%)** | **Median (IQR)** |
| --- | --- | --- |
| **Satisfaction with capacity for work** |  |  |
| Extremely dissatisfied | 8 (1.4%) | 4.0 (3.0 to 4.0) |
| Dissatisfied | 36 (6.4%) | 4.0 (3.0 to 4.0) |
| Neither satisfied nor dissatisfied | 95 (16.9%) | 4.0 (3.0 to 4.0) |
| Fair | 4 (0.7%) | 4.0 (3.0 to 4.0) |
| Satisfied | 310 (55.2%) | 4.0 (3.0 to 4.0) |
| Very satisfied | 109 (19.4%) | 4.0 (3.0 to 4.0) |
| **Satisfaction with work-life balance** |  |  |
| Extremely dissatisfied | 36 (6.4%) | 2.0 (2.0 to 4.0) |
| Dissatisfied | 102 (18.1%) | 2.0 (2.0 to 4.0) |
| Neither satisfied nor dissatisfied | 150 (26.7%) | 2.0 (2.0 to 4.0) |
| Fair | 14 (2.5%) | 2.0 (2.0 to 4.0) |
| Satisfied | 231 (41.1%) | 2.0 (2.0 to 4.0) |
| Very satisfied | 29 (5.2%) | 2.0 (2.0 to 4.0) |
| **Satisfaction with work environment** |  |  |
| Extremely dissatisfied | 44 (7.8%) | 2.0 (2.0 to 4.0) |
| Dissatisfied | 98 (17.4%) | 2.0 (2.0 to 4.0) |
| Neither satisfied nor dissatisfied | 153 (27.2%) | 2.0 (2.0 to 4.0) |
| Fair | 3 (0.5%) | 2.0 (2.0 to 4.0) |
| Satisfied | 216 (38.4%) | 2.0 (2.0 to 4.0) |
| Very satisfied | 48 (8.5%) | 2.0 (2.0 to 4.0) |
| **Satisfaction with professional relationships** |  |  |
| Extremely dissatisfied | 8 (1.4%) | 4.0 (4.0 to 4.0) |
| Dissatisfied | 25 (4.4%) | 4.0 (4.0 to 4.0) |
| Neither satisfied nor dissatisfied | 96 (17.1%) | 4.0 (4.0 to 4.0) |
| Fair | 3 (0.5%) | 4.0 (4.0 to 4.0) |
| Satisfied | 311 (55.3%) | 4.0 (4.0 to 4.0) |
| Very satisfied | 119 (21.2%) | 4.0 (4.0 to 4.0) |
| **Satisfaction with administrative support and recognition** |  |  |
| Extremely dissatisfied | 29 (5.2%) | 4.0 (2.0 to 4.0) |
| Dissatisfied | 63 (11.2%) | 4.0 (2.0 to 4.0) |
| Neither satisfied nor dissatisfied | 140 (24.9%) | 4.0 (2.0 to 4.0) |
| Fair | 6 (1.1%) | 4.0 (2.0 to 4.0) |
| Satisfied | 251 (44.7%) | 4.0 (2.0 to 4.0) |
| Very satisfied | 73 (13%) | 4.0 (2.0 to 4.0) |
| **Satisfaction with training and professional development opportunities** |  |  |
| Extremely dissatisfied | 45 (8%) | 3.0 (2.0 to 4.0) |
| Dissatisfied | 71 (12.6%) | 3.0 (2.0 to 4.0) |
| Neither satisfied nor dissatisfied | 164 (29.2%) | 3.0 (2.0 to 4.0) |
| Fair | 13 (2.3%) | 3.0 (2.0 to 4.0) |
| Satisfied | 223 (39.7%) | 3.0 (2.0 to 4.0) |
| Very satisfied | 46 (8.2%) | 3.0 (2.0 to 4.0) |
| **Overall life satisfaction** |  |  |
| Extremely dissatisfied | 6 (1.1%) | 4.0 (4.0 to 4.0) |
| Dissatisfied | 24 (4.3%) | 4.0 (4.0 to 4.0) |
| Neither satisfied nor dissatisfied | 72 (12.8%) | 4.0 (4.0 to 4.0) |
| Fair | 8 (1.4%) | 4.0 (4.0 to 4.0) |
| Satisfied | 317 (56.4%) | 4.0 (4.0 to 4.0) |
| Very satisfied | 135 (24%) | 4.0 (4.0 to 4.0) |
| **Current job satisfaction** |  |  |
| Extremely dissatisfied | 12 (2.1%) | 4.0 (2.0 to 4.0) |
| Dissatisfied | 41 (7.3%) | 4.0 (2.0 to 4.0) |
| Neither satisfied nor dissatisfied | 105 (18.7%) | 4.0 (2.0 to 4.0) |
| Fair | 9 (1.6%) | 4.0 (2.0 to 4.0) |
| Satisfied | 310 (55.2%) | 4.0 (2.0 to 4.0) |
| Very satisfied | 85 (15.1%) | 4.0 (2.0 to 4.0) |

*Note. Values are presented as N (%) for each response category, with the item-level median (IQR) on its recoded ordinal scale shown in the final column. IQR = interquartile range.*
